# Survival Advantage of Upfront Surgery for Pancreatic Head Cancer Without Preoperative Biliary Drainage

**DOI:** 10.3389/fonc.2020.526514

**Published:** 2020-11-03

**Authors:** Rupaly Pande, James Hodson, Ravi Marudanayagam, N. Chatzizacharias, Bobby Dasari, Paolo Muiesan, Robert P. Sutcliffe, Darius F. Mirza, John Isaac, Keith J. Roberts

**Affiliations:** ^1^ Department of HPB and Liver Transplant Surgery, University Hospitals Birmingham NHS Foundation Trust, Birmingham, United Kingdom; ^2^ Institute of Translational Medicine, University Hospitals Birmingham NHS Foundation Trust, Birmingham, United Kingdom; ^3^ Institute of Immunology and Immunotherapy, University of Birmingham, Birmingham, United Kingdom

**Keywords:** pancreatic surgery, survival, fast track surgery, preoperative biliary drainage, intention to treat (ITT) analysis

## Abstract

**Introduction:**

Level 1 evidence from randomized trials demonstrates less complication when jaundiced patients with resectable pancreatic cancer proceed directly to surgery, rather than undergo preoperative biliary drainage (PBD) first. Although “fast track” surgery significantly increases the resectability rate, it is unknown whether this translates into a survival benefit. This study evaluated the effect of upfront surgery on long-term survival using an intention-to-treat (ITT) analysis.

**Methods:**

Patients were identified from a prospectively maintained database, stratified according to whether or not they underwent PBD.

**Results:**

Among 157 patients, 84 (54%) underwent PBD. Of these, 73% underwent surgery, compared to 100% of those without PBD (p<0.001). Reasons for not undergoing surgery were progression of cancer (N=11), progressive frailty (N=5), or PBD-related complication (N=7). In those who underwent surgery, PBD was associated with a longer time from diagnosis to surgery (median: 59 vs. 14 days, p<0.001), and a higher rate of unresectable cancer at surgery (26% vs. 3%, p<0.001). On an ITT basis, patients treated with PBD had significantly shorter survival, at a median of 15 vs. 19 months (HR: 1.59, 95% CI: 1.07–2.37, p=0.023). However, for the subset of patients who underwent resection, survival was similar in the two groups (HR: 1.07, 95% CI: 0.66–1.73, p=0.773).

**Conclusions:**

A reduced time to surgery with avoidance of PBD offers survival benefit. This is only appreciated on ITT analysis, which includes patients who are initially considered candidates for surgery, but ultimately do not undergo surgery. Considering this ‘hidden’ cohort of patients is important when considering optimal pathways for the treatment of resectable pancreatic cancer.

## Introduction

Survival for pancreatic cancer remains bleak, with five year survival rates of just 1%–4%, and overall survival remaining largely unchanged over the last 40 years (1, 2). Contrasted with the laudable rise in life expectancy for the general population (3), the subsequent expected years lost following pancreatic cancer presents a startling gravity of the burden of disease. Survival is therefore an important measure of success of our management of the disease (4). The three impediments to survival are thought to be tumor biology (5), rapidity of diagnosis and management (6, 7), and efficacy of treatment (8, 9).

Progression of pancreatic cancer is known to be aggressive and rapid (10), with the propensity to invade adjacent structures and metastasize, rendering patients inoperable by the time of surgery. Currently, surgery affords the only potential for cure (11); hence efficient diagnosis and management is of paramount importance. Prolonged time to curative resection in solid tumors such as colorectal, lung, breast and bladder has been shown to adversely affect tumor stage and survival (12), but studies have not successfully demonstrated the same effect in pancreatic cancer (13–15). The pathway to surgery among patients with pancreatic head cancer is complicated by the presence of obstructive jaundice in the majority of cases. Preoperative biliary drainage (PBD) has remained the standard of care in many regions, despite clear evidence of the harm associated with this procedure (16–18).

A direct-to-surgery approach for patients with jaundice thus avoids the harm of PBD and, by necessity, requires a short time from presentation to surgery. Increasing time to surgery is associated with a reduction in the rates of resectability. However, the impact of a direct-to-surgery approach upon long term survival has yet to be shown.

This study aims to assess the impact of a rapid pathway with the avoidance of PBD upon survival among jaundiced patients with pancreatic cancer.

## Methods

The study was conducted in line with the STROBE (Strengthening the Reporting of Observational studies in Epidemiology) guidelines (19) and was approved by the local audit committee. This study was based at the University Hospitals Birmingham (UHB), a tertiary referral centre serving a population of around 4 million. UHB is a specialist centre for the treatment of pancreatic cancer, with patients either presenting directly, or being referred from a non-specialist centre within the catchment area. Patients initially receive a diagnostic CT scan, which is then discussed by a multidisciplinary team, who meet weekly to assess new referrals. Since August 2015, UHB has used a “fast track” pathway for the treatment of pancreatic cancer, which prioritizes early surgery, while avoiding PBD, and has previously been described in detail elsewhere (20). Patients with renal dysfunction not easily correctable with a short duration of fluid replacement therapy or with biliary sepsis or complete occlusion of SMV/PV are considered for stenting with or without neoadjuvant chemotherapy (NAT).

For this study, consecutive patients referred to the pancreatic cancer team between August 2015 and December 2017 were identified. Data were obtained from hospital electronic records to include demographic, clinical, radiological and histological factors, as well as patient survival. The AJCC 8^th^ edition was used for TNM staging. The inclusion criteria of the study were diagnoses of both pancreatic cancer (either suspected or confirmed) and jaundice (defined as serum bilirubin in excess of 30µmol/l), as well as fitness for surgery (WHO performance status 0 or 1) at the time of referral. Only patients where the pancreatic cancer was deemed to be potentially resectable at referral were included in the study. This was defined as a tumor with or without venous involvement, where any venous disease was considered to be resectable and could be reconstructed with or without using a vein graft. Exclusion criteria were patients with borderline or locally advanced (as per NCCN criteria) (21), arterial involvement, or where they were treated with neoadjuvant chemotherapy.

Patients received standard adjuvant chemotherapy, which was gemcitabine for the majority of the study period. Towards the end of the study, with the publication of the ESPAC 4 study (European Study Group for Pancreatic Cancer) (22), gemcitabine and capecitabine became standard therapy.

For analysis, patients were divided into two groups, based on whether or not PBD had been used. Analyses were performed on an intention-to-treat (ITT) basis with respect to surgery, and included all patients initially considered suitable for potentially curative resection surgery, regardless of whether or not such surgery was performed. The primary endpoint was overall survival, defined as the time from the initial diagnostic CT to the date of death or last follow up.

### Statistical Methods

Initially, comparisons were made between patients with and without PBD. Continuous factors were reported as mean ± standard deviation (SD) if normally distributed, and medians and interquartile ranges (IQRs) otherwise, with comparisons between groups made using Mann-Whitney U tests. Ordinal factors were also analyzed using Mann-Whitney U tests, with Fisher’s exact tests used for nominal factors. Similar analyses were also performed to compare those that underwent surgery to those that did not. Patient survival was analyzed using Kaplan-Meier curves, with univariable Cox regression models used to generate hazard ratios and p-values.

All analyses were performed using IBM SPSS 22 (IBM Corp. Armonk, NY), with p<0.05 deemed to be indicative of statistical significance throughout.

## Results

### Patient Demographics

A total of 157 patients met the inclusion criteria of the study, of whom 84 (54%) underwent PBD, of which there were 14 PTC, 51 SEM, 19 were plastic stents. The number of PBDs was recorded in 72 of these, with 71% having a single PBD, and 18%, 7%, and 4% receiving 2, 3, and 4 interventions, respectively. Of those receiving PBDs, these were inserted prior to referral in 77 (92%) of cases. Of these, there was only a specific clinical indication in 4 (5%) patients, namely acute kidney injury (N=2) and sepsis (N=2). In the N=7 (8%) patients with PBDs inserted after referral, the decision to perform the intervention was made by the MDT team. Stenting was performed in these patients due to diagnostic uncertainty (N=4) and anaesthetic work up (N=3).

Comparisons between patients that did and did not undergo PBD are reported in **Table 1**. This found the demographics of the two groups to be similar, with no significant differences detected in patient age, gender, BMI, CCI, smoking status, or CA19-9 levels.

**Table 1:** Patient demographics and surgical factors by PBD.

		PBD		
	N	*No (n=73)*	*Yes (n=84)*	p-Value
***Patient Demographics***				
Age at MDT Assessment (Years)	157	66.2 ± 8.7	68.9 ± 9.7	0.075
Gender (% Male)	157	36 (49%)	47 (56%)	0.427
BMI	152	26.7 ± 4.9	27.4 ± 5.5	0.152
CCI	157			0.199*
*2-3*		14 (19%)	15 (18%)	
*4-5*		41 (56%)	38 (45%)	
*6+*		18 (25%)	31 (37%)	
Smoking Status	157			0.286
*No*		60 (82%)	71 (85%)	
*Current*		10 (14%)	6 (7%)	
*Ex*		3 (4%)	7 (8%)	
CA19-9 (U/ml)	135	307 (116–959)	318 (92–1718)	0.643
Surgery	157	73 (100%)	61 (73%)	**<0.001**
***Surgical Factors***				
Days from Diagnosis to Surgery**	134	14 (10–21)	59 (45–77)	**<0.001**
Bilirubin (at Surgery, µmol/L)**	133	307 (222–411)	15 (8–61)	**<0.001**
Type of Surgery**	134			**<0.001**
*Resection*		71 (97%)	45 (74%)	
*Bypass*		2 (3%)	16 (26%)	
Vein Reconstruction***	116	20 (28%)	8 (18%)	0.292
T-Stage***	116			0.496*
*T1*		13 (18%)	9 (20%)	
*T2*		55 (77%)	30 (67%)	
*T3*		3 (4%)	6 (13%)	
N-Stage***	116			0.547*
*N0*		9 (13%)	4 (9%)	
*N1*		32 (45%)	20 (44%)	
*N2*		30 (42%)	21 (47%)	
Overall Stage***	116			0.468*
*1*		8 (11%)	3 (7%)	
*2*		32 (45%)	20 (44%)	
*3*		31 (44%)	22 (49%)	
R-Status (% R1)***	116	29 (41%)	17 (38%)	0.846
LN ratio***	116	0.19 (0.07–0.36)	0.21 (0.06–0.33)	0.966

Patient demographics are reported for the cohort as a whole, while surgical factors are reported only for the subgroup who underwent surgery (N=73/61 for PBD No/Yes), and tumor staging and resection-related factors for the subgroup that underwent resection (N=71/45). Continuous data are reported as mean ± SD, or as median (interquartile range), with p-values from Mann-Whitney U tests. Categorical data are reported as N (%), with p-values from Fisher’s exact tests or Chi-square. Bold p-values are significant at p<0.05. *p-Value from a Mann-Whitney U test, as the factor is ordinal. **In patients undergoing surgery; time is from initial CT scan. ***In patients undergoing resection.

MDT, multidisciplinary team; BMI, body mass index; CCI, Charlson Comorbidity Index; LN ratio, lymph node ratio.

### Surgical Approach

Only 73% of patients with PBD underwent surgery, compared to 100% of those without PBD (p<0.001). Patients treated surgically were found to be significantly less comorbid (p<0.001), and to have a significantly lower CA19-9 (p=0.006, **Table 2**). For those who underwent surgery, PBD was associated with a significantly lower pre-operative bilirubin level, as would be expected, with a median of 15 vs. 307 µmol/L (p<0.001, **Table 1**). However, patients undergoing PBD before surgery also had a significantly longer time from diagnosis to surgery (median: 59 vs. 14 days, p<0.001), as well as a significantly lower resection rate (74% vs. 97%, p<0.001). For those that were resected (N=116), no significant differences in the T/N-staging, R-status or LN ratio were detected between those patients with and without PBD.

**Table 2:** Patients demographics by surgery.

		Surgery		
	N	*No (n=23)*	*Yes (n=134)*	p-Value
***Patient Demographics***				
Age at MDT Assessment (Years)	157	70.8 ± 11.1	67.1 ± 8.9	0.057
Gender (% Male)*	157	16 (70%)	67 (50%)	0.113
BMI	152	27.3 ± 7.3	27.0 ± 4.9	0.642
CCI*	157			**<0.001**
*2-3*		1 (4%)	28 (21%)	
*4-5*		8 (35%)	71 (53%)	
*6+*		14 (61%)	35 (26%)	
Smoking Status*	157			0.420
*No*		18 (78%)	113 (84%)	
*Current*		4 (17%)	12 (9%)	
*Ex*		1 (4%)	6 (7%)	
CA19-9 (U/ml)	135	1444 (314–4219)	280 (94–959)	**0.006**

Continuous data are reported as mean ± SD, or as median (interquartile range), with p-values from Mann-Whitney U tests. Categorical data are reported as N (%), with p-values from Fisher’s exact tests or Chi-square. Bold p-values are significant at p < 0.05. *p-Value from a Mann-Whitney U test, as the factor is ordinal.

### Survival From Diagnosis

**Figure 1:**
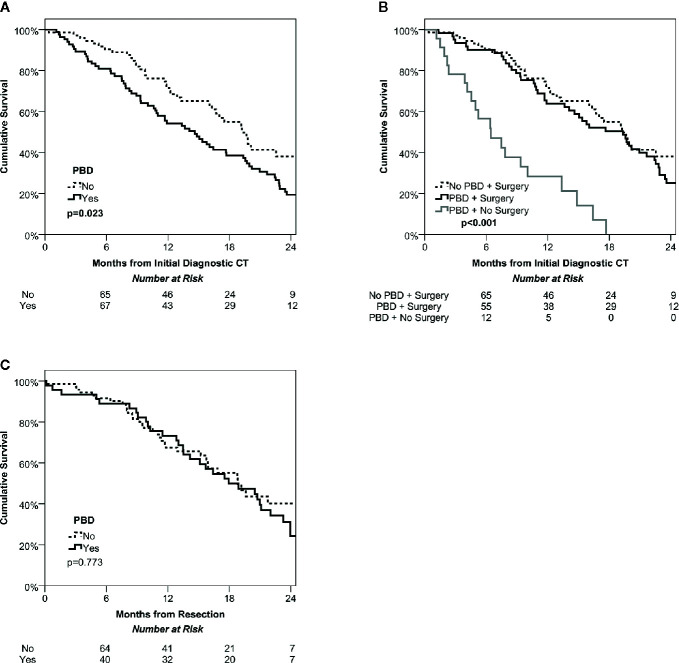
Kaplan-Meier curves of overall survival among the study cohort. Intention to treat survival among the whole cohort stratified by management of jaundice (PBD vs no PBD) demonstrates a significant survival benefit of ‘fast track’ surgery avoiding PBD **(A)**. Dividing the PBD group into those that did and did not undergo surgery found similar survival in the PBD and no PBD cohorts that were treated surgically, but poor survival in the non-surgical group **(B)**. Overall survival after potentially curative resection was also similar in the PBD and no PBD groups **(C)**.

Over a median follow up time of 14 months (IQR: 8-20) from the diagnostic CT, there were a total of N=107 deaths, giving Kaplan-Meier estimated survival rates of 85%, 62% and 26% at 6, 12, and 24 months, respectively. Patients undergoing PBD were found to have significantly shorter survival than the non-PBD group (p=0.023, **Figure 1A**), with medians of 15 vs. 19 months and a hazard ratio of 1.59 (95% CI: 1.07–2.37). This was largely due to the significantly lower rate of surgery in the PBD group. For the subgroup of patients where surgery was performed, survival was similar in PBD and non-PBD groups, with both having a median of 19 months (HR: 1.22, 95% CI: 0.79–1.87, p=0.369). However, those patients who underwent PBD but did not receive surgery had significantly shorter survival then either of the surgical groups, with a median of only 6 months (both p<0.001, **Figure 1B**).

### Survival From Resection

Analyses were then performed on the subgroup of patients who received resections, of whom N=45 underwent PBD, and N=71 did not. Comparisons between these groups found no significant difference in post-resection survival (p=0.773, **Figure 1C**), with medians of 18 vs. 19 months in those with vs. without PBD, giving a hazard ratio of 1.07 (95% CI: 0.66–1.73).

### Reasons for Patients Not Receiving Curative Surgery

Some 27% (N=23) of the PBD cohort did not undergo surgery, most commonly due to progression of either cancer (N=11) or the frailty of the patient (N=5, **Table 3**). However, there were also seven patients where the primary reason for not receiving curative surgery was due to complications of PBD. Of those patients that received surgery after PBD, 26% (N=16) were found to have unresectable disease, and so were treated with a bypass.

**Table 3:** Reasons for initially resectable patients not undergoing surgery after PBD.

**Progression of cancer (N=11)**
Development of metastases (+/- local progression) on repeat CT scan associated with pathway delays (N=11)
**Progression of frailty (N=5)**
Developed a non-biliary infection, and failed to recover to a level of fitness for surgery (N=2)
Deteriorated, and became too frail for surgery (N=2)
Needed cardiac valve replacement, relisted but progressed (N=1)
**PBD related complication (N=7)**
Developed cholangitis, and then died (N=3)
Developed cholangitis followed by disease progression and death (N=1)
Developed renal failure, and then died (N=1) Deteriorated due to renal failure after PBD, and became too frail for surgery (N=1)
Developed pancreatitis, and then died (N=1)

## Discussion

This was an intention-to-treat analysis of survival among patients with jaundice and resectable pancreatic cancer, based on initial assessments of radiologic and physiologic suitability for surgery. The main finding of this study was that patients proceeding directly to surgery without PBD had significantly longer survival than those treated with PBD. The difference in survival was largely attributable to the surprisingly large proportion of patients who never underwent surgery after PBD (27%), who subsequently survived for a median of only 6 months. For these patients, the most common reason for not progressing with surgery was due to disease progression on repeat imaging (11/23). This is an important observation, as present NICE guidelines suggest that patients should undergo PET CT to fully stage their disease. As such, whilst it is highly likely that some patients in the upfront surgery group harbored occult metastatic disease which could have been identified by PET scan, the delays to surgery in the PBD group, which are in large part due to multiple diagnostic and staging investigations, may have contributed to disease progression, the very thing they are meant to define.

There are thus two major conclusions drawn from this work. The first is that PBD, and the resulting slow pathway to surgery, is associated with significantly shorter patient survival. *However, importantly, this is not apparent when the outcome of patients undergoing surgery is considered. The effect is only observed on intention-to-treat analysis, which includes all patients who begin the treatment pathway*. The second is that clinicians need to rationalize staging investigations to prevent delays to treatment, or provide them within a suitably rapid pathway. It is known from other work that a small proportion of patients have occult disease at presentation. There is thus a need to consider which pathway represents the most overall benefit for patients. It may be desirable to perform MRI and/or PET imaging to attempt to diagnose patients with occult disease, but the very process of making the diagnostic pathway more complex adds time and may mean that more patients require PBD. It is to be noted that pathways in this organisation are relatively simple; others can include laparoscopy or formal testing of physiology, which could further complicate the pathway. The time to surgery in the PBD cohort is longer than what is typically reported but not without precedent. Recent studies from France (23) and Sweden (7) have reported median times to surgery between and 42 and 64 days. Importantly, a recent meta-analysis (24) demonstrated a link between increased time to surgery and reduced resection rate. It may be that the system of centralized surgery in the United Kingdom has fragmented and made the pathway to surgery more complex consequently increasing the time to surgery. However, benefits of centralisation have been realized in terms of reduced perioperative mortality (25).

This is an observational study and, thus, there will be risk of selection bias. The main criticism could be that there are reasons why the present PBD cohort underwent biliary drainage, and that those reasons would, in turn, be associated with poor prognosis. However, it can be seen from the data that the vast majority of PBD were performed prior to referral to our service. Had these patients been referred earlier, then they would have been offered early surgery avoiding PBD.

There are advocates of neoadjuvant therapy for resectable pancreatic cancer. Certainly, excellent outcomes can be observed when patients undergo this treatment for borderline or locally advanced tumors; low rates of margin positivity, nodal involvement and encouraging duration of survival can be seen (26). However, there remain three fundamental problems with this approach for patients with *resectable* pancreatic cancer. Firstly, jaundiced patients must undergo PBD to receive neoadjuvant therapy, thus exposing them to potentially avoidable harm, which is clearly quantified is this study and elsewhere (16). Secondly, the majority of patients undergoing neoadjuvant therapy receive FOLFIRINOX, as most patients’ tumors progress on therapy with other regimens; this limits the ability to generalize the potential benefit of neoadjuvant therapy, as most elderly or frail patients are unlikely to receive this therapy. Finally, the timing of therapy—neoadjuvant vs adjuvant—is likely to be less important than which therapy is provided. The excellent outcomes of neoadjuvant therapy with FOLFIRINOX are mirrored by the remarkable survival among patients receiving adjuvant therapy with FOLFIRINOX, as reported by Conroy et al. (27).

Thus, the devil is in the detail. Most articles that report survival of adjuvant or neoadjuvant therapy do not include those patients initially considered as resectable, but who ultimately fail to undergo surgery; results therefore over-report survival outcomes. If data from this intention-to-treat study is compared with a recent meta-analysis by Versteijne et al, the experience of the upfront surgery cohort in this study (median survival: 19 months) compares favorably with other cohorts undergoing upfront surgery for resectable cancer (17.7 months) (28). This is particularly noteworthy, as every patient in this study within the upfront surgery pathway underwent surgery. The problems with selection bias can be seen clearly within this work. Within the PBD group, those that underwent surgery (including those with bypass surgery) achieved a similar survival to the upfront surgery group, with a median of 19 months in both groups. However, inclusion of the cohort that never achieved surgery reduced the median survival by 21% to 15 months. These survival data need to also be considered in the context of what adjuvant therapy was provided. Most patients will have received single agent gemcitabine, as results from the ESPAC4 study were available only towards the end of the study, and data from PRODIGE-24 (Partenariat de Recherche en Oncologie Digestive 24) had not been published.

This study has some limitations. The primary one being that this was a non-randomized observational study. A further main limitation is that, of the patients that initially presented to other hospitals, only those that were eventually referred to the specialist centre for treatment would have been identified for inclusion in the study. As such, any patients that died soon after presentation, but prior to referral, would have been excluded from the study, potentially introducing some degree of survivorship bias into the analysis. As PBD was mostly used in those presenting to other hospitals, this bias would likely result in an overestimate of the survival time in patients treated with PBD. The second limitation related to the use of the diagnostic CT scan as the start of follow up in the primary survival analysis. This was a reasonable date to use for patients that presented at the specialist centre, as it would be an accurate representation of the date of diagnosis. However, for patients initially presenting to other centres, treatment may have commenced prior to referral. As a result, using the date of the diagnostic CT scan would underestimate survival time in these patients, particularly in those harmed by PBD, where the time to referral would have been longer. However, since patients are generally referred quickly, generally within days or weeks of presentation, the impact of this limitation on the analysis should have been minimal.

In summary, a direct-to-surgery approach for resectable pancreatic cancer improves survival by avoiding harm done by PBD or delays to treatment and apparent disease progression on repeat imaging. Furthermore, this strategy reduces cost and uses of hospital resources and of patient discomfort, even when no significant harm is done, associated with PBD. Efforts to standardize this approach, optimize patient recovery and well-being and subsequent likelihood of receiving adjuvant therapy, FOLFIRINOX where possible, should improve survival and patient experience among those with resectable pancreatic cancer.

## Data Availability Statement

The datasets generated for this study are available on request to the corresponding author.

## Ethics Statement

The University Hospitals Birmingham NHS Trust provided institutional approval to review patients’ data and report outcomes for this study.

## Author Contributions

RP drafted the manuscript, organized and analyzed the database, and interpreted the data. JH organized the database and provided the statistical analysis. RM, NC, BD, and PM revised the manuscript critically for important intellectual content. RS contributed to the conception and revised the manuscript critically for important intellectual content. JI contributed to the conception of the study. KR contributed to the conception and design of the study and revised the manuscript critically for important intellectual content. All authors contributed to the article and approved the submitted version.

## Conflict of Interest

The authors declare that the research was conducted in the absence of any commercial or financial relationships that could be construed as a potential conflict of interest.
